# Superantigens: Molecular Basis for Their Role in Human Diseases

**DOI:** 10.3201/eid1405.080089

**Published:** 2008-05

**Authors:** D. Scott Schmid

**Affiliations:** * Centers for Disease Control and Prevention, Atlanta, GA, USA

**Keywords:** Antigens, human, diseases, book review

This collection of short reviews by experts in the field provides a complete overview of microbial superantigens, an unusual family of proteins that form an abnormal linkage between the major histocompatibility complex class II antigens and specific T-cell repertoire Vβ families. This linkage leads to the nonspecific activation of large numbers of regulatory T lymphocytes, producing cytokine storms that can have a variety of serious clinical consequences. 

The book is organized into 5 sections with a total of 16 chapters. The first section is an overview of the breadth and scope of superantigen research, including an up-to date catalog of superantigens characterized from both bacteria and viruses, their cellular interactions, and disease associations. The next 3 chapters deal with the 3-dimensional structure, function, and diversity of superantigens, including an account of the critical involvement of zinc in the optimal binding of some of these proteins. Section 3 contains an entire chapter that describes the pathophysiology of superantigens in both acute and chronic skin disorders. Several chapters in section 4 describe in vitro and animal model systems for the study of diseases caused by superantigens, including autoimmune disease, neuropathology, toxic shock, and others. 

The final 4 chapters in section 5 detail various therapeutic approaches for superantigen-mediated diseases. These approaches include conventional antibiotics, antagonistic peptides, intravenous immunoglobulin, antibodies directed to T-cell costimulatory receptors, and superantigen receptor mimics, in addition to existing and experimental approaches. An unnumbered section after the first chapter contains high-quality color plate illustrations, which collectively provide outstanding visual support for several chapters.

Superantigens affords a comprehensive look at the current state of knowledge regarding these interesting proteins in a relatively compact volume. The text is certainly a must-read for any scientist engaged in their study but will also prove a rewarding read for microbiologists interested in this curious interaction between mibrobes and the immune system.

